# Advanced Acral Melanoma Therapies: Current Status and Future Directions

**DOI:** 10.1007/s11864-022-01007-6

**Published:** 2022-09-20

**Authors:** Yiqun Zhang, Shijie Lan, Di Wu

**Affiliations:** Cancer Center, The First Hospital of Jilin University, 1 Xinmin St, Changchun, 130021 China

**Keywords:** Acral melanoma, Targeted therapy, Immunotherapy, Combination therapy

## Abstract

Melanoma is one of the deadliest malignancies. Its incidence has been significantly increasing in most countries in recent decades. Acral melanoma (AM), a peculiar subgroup of melanoma occurring on the palms, soles, and nails, is the main subtype of melanoma in people of color and is extremely rare in Caucasians. Although great progress has been made in melanoma treatment in recent years, patients with AM have shown limited benefit from current therapies and thus consequently have worse overall survival rates. Achieving durable therapeutic responses in this high-risk melanoma subtype represents one of the greatest challenges in the field. The frequency of BRAF mutations in AM is much lower than that in cutaneous melanoma, which prevents most AM patients from receiving treatment with BRAF inhibitors. However, AM has more frequent mutations such as KIT and CDK4/6, so targeted therapy may still improve the survival of some AM patients in the future. AM may be less susceptible to immune checkpoint inhibitors because of the poor immunogenicity. Therefore, how to enhance the immune response to the tumor cells may be the key to the application of immune checkpoint inhibitors in advanced AM. Anti-angiogenic drugs, albumin paclitaxel, or interferons are thought to enhance the effectiveness of immune checkpoint inhibitors. Combination therapies based on the backbone of PD-1 are more likely to provide greater clinical benefits. Understanding the molecular landscapes and immune microenvironment of AM will help optimize our combinatory strategies.

## Introduction

Melanoma is classified into four major subtypes: skin melanomas without chronic sun-induced damage; skin melanomas with chronic sun-induced damage; mucosal melanomas; acral melanomas [1]. Acral melanoma (AM) is rare in Caucasians (1–7%) [2–5] but has a higher incidence in non-White individuals, accounting for up to 50–58% of all melanomas in Asians [6] and even more (60–70%) in Blacks [7].

Although AM causes a large number of deaths in Europe and the USA and exhibits unique clinical and biological characteristics, due to its relative rarity, there are few related studies, and it is often overlooked. Hence, the understanding of AM is still limited. Herein, we reviewed the therapeutic status of AM and proposed potential therapeutic strategies based on its genomics and tumor immune microenvironment characteristics.

## Current status

### Chemotherapy

For patients with metastatic melanoma, dacarbazine was the most commonly used chemotherapy drug for decades but its efficacy was not ideal [8–11]. Besides failed dacarbazine-based therapy, very little is known about salvage chemotherapy in metastatic AM patients. For example, a clinical trial in China confirmed the efficacy and safety of albumin paclitaxel + carboplatin to treat metastatic melanoma. This study showed that the disease control rate (DCR) in AM patients was 81.3%, the median progression-free survival (PFS) was 6 months, and the median overall survival (OS) was 17 months [12]. Besides this study, there are few studies regarding other chemotherapy drugs to treat AM.

### Immunotherapy

#### Immune checkpoint inhibitors

Since 2011, many immune checkpoint inhibitors (ICIs) have been approved by relevant authorities in the USA, Europe, and China for melanoma treatments. These drugs mainly include ipilimumab, pembrolizumab, nivolumab, and torpalimab [13]. Notably, AM patients respond much worse to ICIs than CM patients [14–17].

Nivolumab, pembrolizumab, and torpalimab monotherapy can lead to better clinical outcomes in advanced AM patients compared to ipilimumab [18••]. Previous studies have shown that for advanced AM patients who received first-line treatment with ipilimumab, the objective response rate (ORR) was 17.8%, the median PFS was 6.9 months, and the median OS was 38.7 months [19]. For advanced AM patients who received post-line treatment with ipilimumab, the ORR was 11.4%, the median PFS was 2.5 months, and the median OS was 7.1–16.7 months [20, 21]. In contrast, advanced AM patients who received first-line anti-PD-1 monotherapy had an ORR of 34.0–40.0%, median PFS of 3.1–9.2 months, and median OS of 18.6–60.1 months [19, 22]. Advanced AM patients who received post-line therapy with anti-PD-1 monotherapy had an ORR of 14.0–32.0%, median PFS of 3.2–4.1 months, and median OS of 16.9–25.8 months [17, 22–27].

Notably, AM patients at different primary sites might respond differently to ICIs. For example, one retrospective study in Japan with 193 advanced AM patients who received nivolumab or pembrolizumab (nail apparatus = 70; palm and sole = 123), of which 143 were first-line treatments, showed that the ORRs of the palm and sole group and the nail apparatus group were 21.1 and 8.6%, respectively, and the median OS were 22.3 and 12.8 months, respectively [28•].

#### Oncolytic virus

Talimogene laherparepvec (T-VEC) is an oncolytic virus that induces tumor-specific T-cell responses via reduction of virally mediated suppression of antigen presentation, stimulation of viral pathogenicity, and enhancement of tumor-selective replication [29–31]. Recent case studies have confirmed the efficacy of this drug in AM patients [32].

Additionally, a phase II clinical trial evaluated the safety and efficacy of OrionX010 (a herpes simplex virus type I oncolytic virus) with 26 unresectable melanoma patients in China, of which 18 (69.2%) were AM patients. In this trial, AM patients presented a median PFS of 3.0 months and a median OS of 19.2 months [33•].

#### Imiquimod

Imiquimod is a Toll-like receptor 7 (TLR7) agonist. It promotes the induction of CD4^+^ T cells and the antitumor response of CD8^+^ T cells by activating TLR7 located on antigen-presenting cells and shifts the immune response to a direction mediated by the T helper 1 (Th1) cells [34, 35]. A retrospective analysis, which included 20 cases of melanoma patients (AM=10) with locoregional cutaneous metastases of melanoma (LCMM), evaluated the response of LCMM to cryotherapy combined with 5% imiquimod local treatment. Regarding locoregional response, 13 patients (65%) responded to treatment, eight (40%) of these completely and five (25%) partially. In assessing overall response, three patients (15%) had complete response and one patient (5%) had stable disease [36].

### Molecular targeted therapy

The efficacy of targeted therapy in AM patients has been previously demonstrated. The main signaling pathways known as abnormal during AM onset include MAPK, PI3K/AKT/PTEN, JAK/STAT3, MDM2/TP53, WNT, MCR1-MITF, TERT, and WNT/CDK4/CDKN2A [37]. To date, approximately half (42–55%) of AMs studied have BRAF, RAS, or NF1 mutations, besides triple-wild-type (TWT) mutations [38]. TWT driver mutations include genetic alterations in various genes, such as KIT, CCND1, CTNNB1, KDR(VEGFR2), MDM2, BCL2, AKT3, IDH1, GNAS, CDK4, CDKN2A, MITF, PTEN, RB1, TP53, APC, ERBB2, ERBB3, NUAK2, ABCB5, and TERT [37–45].

#### BRAF/MEK inhibitors

The frequency of BRAF mutations in AM is low (only 15–20%) [46–49], which limits the use of BRAF inhibitors in AM patients. Common BRAF inhibitors include vemurafenib, dabrafenib, and encorafenib [50•]. Recently, 20 Chinese AM patients with a BRAF mutation that received vemurafenib presented an ORR of 69.2%, a median PFS of 5.4 months, and a median OS of 11.7 months [51].

The combination of BRAF and MEK inhibitors has been widely used to treat BRAF-mutant melanoma patients, achieving satisfactory results [52]. The current MEK inhibitors used to treat melanoma mainly include trametinib, cobimetinib, and bindetinib [53]. For example, a phase II clinical trial in China observed long-term survival outcomes for unresectable or metastatic acral/cutaneous melanoma patients, including 12 AM patients who presented an ORR of 83.3% and a 3-year OS of 35.7% [54•]. Another retrospective analysis included 112 advanced melanoma patients (11 AM and 3 mucosal melanoma patients) who received a combination of BRAF and MEK inhibitors. In this study, AM and mucosal melanoma patients presented an ORR of 64.3% [55].

#### KIT inhibitors

KIT mutations and/or amplifications are more common in AM than those in other melanoma types (10–20%) [56, 57]. Currently, common KIT inhibitors include imatinib, sunitinib, dasatinib, and nilotinib [58], but only imatinib and nilotinib are effective in AM. A phase II clinical trial in China evaluating the effectiveness of imatinib in 43 metastatic melanoma patients (AM = 21) harboring c-Kit mutation or amplification showed a median PFS of 3.5 months, a 6-month PFS rate of 36.6%, and DCR of 53.5% [59]. Similarly, a retrospective analysis of 78 metastatic melanoma patients (AM = 42) with c-Kit mutation or amplification treated with imatinib presented median OS and PFS of all patients of 13.1 and 4.2 months, respectively. The ORR and DCR were 21.8% and 60.3%, respectively [60]. Notably, a phase II clinical trial showed that imatinib was ineffective in patients with only KIT amplification (the best overall response rate was 0%) [61]. Moreover, previous phase II clinical studies have shown an ORR of 25–32% and a DCR of 74–80% in advanced AM patients treated with nilotinib [62, 63]. Additionally, previous studies showed that sunitinib and dasatinib were less effective to treat advanced AM patients with KIT mutations [64, 65].

#### CDK4/6 inhibitors

The CDK4/CCND1 mutation and amplification are often present in AM, suggesting that CDK4/6 inhibitors can be used for treatment. In a recent phase II trial, 15 advanced AM patients with genetic aberrations in the CDK pathway were treated with palbociclib. Three (20.0%) patients achieved tumor shrinkage at 8 weeks, including one with confirmed partial response. The median PFS was 2.2 months, and the median OS was 9.5 months [66•]. Additionally, trials with the CDK inhibitor dinaciclib for the treatment of advanced melanoma, including AM, have been completed but the results were not published (NCT00937937).

#### Targeted therapy for NRAS-mutant AM

Currently, there are no drugs that directly target NRAS mutations. NRAS regulates the PI3K/Akt cascade and BRAF activation, resulting in subsequent activation of the MAPK pathway. Therefore, NRAS mutation is a genetic mutation contributing to acquired BRAF inhibitor resistance [67••]. The current treatments of melanoma patients with NRAS mutations are primarily focused on the use of MEK inhibitors to target key signal transduction pathways of the MAPK pathway [68]. Binimetinib was the first MEK inhibitor to show activity in the treatment of NRAS-mutant melanomas, but the effects were not ideal [60, 69, 70]. Clinical trials with the novel MEK inhibitor HL-085 for the treatment of advanced melanoma are being recruited (NCT05217303, NCT05263453, NCT03973151). Additionally, the potential therapeutic value of MEK inhibitors combined with inhibition of downstream effectors (MAPK, PI3K, or CDK4/6) or upstream effectors (RTK, STK19) for melanomas with NRAS mutations has been demonstrated, but the choice remains controversial [71]. There is currently a lack of reports regarding treatments of AM with NRAS mutations.

### Combination therapies

#### Combination of immunotherapies

Interferon-α (IFN-α) stimulates the secretion of IP-10 (CXCL10), recruits effector T cells to the tumor microenvironment, and upregulates the expression of MHC-1 molecules on the surface of tumor cells, thereby enhancing the anti-tumor effects of CD8^+^T cells in the tumor microenvironment [72]. Additionally, IFN-α upregulates the expression of PD-L1 [73]. A retrospective analysis in China suggested that prior therapy with PEG-IFN-α improved the median recurrence-free survival of adjuvant pembrolizumab in resectable advanced melanoma [74•]. These results indicated the feasibility of IFN-α combined with PD-1 inhibitors to treat advanced AM.

Furthermore, CTLA-4 and PD-1 can inhibit anti-tumor immunity through different mechanisms [75]. At the 2021 ESMO Annual Meeting, a retrospective study evaluated the efficacy of combination therapy with PD-1 and CTLA4 inhibitors versus single ICIs in 256 advanced AM patients. Among them, 151, 51, and 54 received anti-PD1, anti-CTLA4, and anti-PD1 and anti-CTLA4 combination therapy, respectively. The median follow-up was 8.1 years, the ORRs were 26, 12, and 44%, respectively, and the median PFS were 7.0, 4.9, and 7.3 months, respectively [76]. The combination of ICIs was superior to ICI alone for ORR, but not for PFS or OS. A phase Ib clinical trial in China (NCT04197882) confirmed the feasibility of the oncolytic virus OrionX010 combined with toripalimab to treat 24 patients with resectable stage IIIB-IVM1a AM. In this trial, 81% of patients showed pathologic responses, and 33% presented radiographic responses. The median follow-up time was 8.9 months and no patient presented recurrence. The recurrence-free survival assessment is ongoing.

#### Combination of chemotherapy and immunotherapy

Temozolomide can enhance the antitumor activity of pembrolizumab by depleting or inhibiting regulatory T cells (Tregs) in the tumor microenvironment [77, 78]. A multicenter retrospective analysis in China with 69 metastatic melanoma patients (28 cases of AM) presented an ORR and median PFS for pembrolizumab plus temozolomide significantly superior to pembrolizumab or temozolomide alone [79•]. These results partly suggested that anti-PD-1 combined with temozolomide can be used as a first-line treatment option for unresectable advanced melanoma, including AM. Another retrospective analysis with 20 advanced AM patients who received treatment with a PD-1 inhibitor plus albumin paclitaxel presented an ORR of 20% and a DCR of 75% [80•].

#### Combination of targeted therapy and immunotherapy

Inhibition of BRAF and MEK can exert immunomodulatory effects and enhance anti-tumor immunity [81–84]. Increased expression of PD-1 and its ligand, PD-L1, has been reported in advanced melanoma patients treated with BRAF inhibitors [85]. Additionally, MEK inhibitors protect tumor-infiltrating CD8^+^ T cells from death caused by T-cell receptors(TCR) stimulation [86]. Besides the IMspire150 trial, the keynote-022 and COMBI-I trials did not demonstrate that compared with targeted drugs, PD-(L)1 monoclonal antibody combined with BRAF and MEK inhibitors increased the PFS in BRAF mutation-positive advanced melanoma patients [87–89]. However, no reports on AM are currently available.

#### Combination of immunotherapy with antiangiogenic therapy

Anti-angiogenic drugs can improve patient response to ICIs by promoting antitumor immunity [90••]. A Chinese clinical trial evaluating the safety and efficacy of camrelizumab combined with apatinib in advanced AM patients is in progress (NCT03955354). Preliminary results showed that among the 27 AM patients, the ORR and DCR were 22.2% and 77.8%, respectively. The median PFS was 8.0 months and the 1-year durable response rate was 83.3%.

#### Combination of chemotherapy with antiangiogenic therapy

The combination of anti-angiogenesis therapy and chemotherapy might produce a synergistic antitumor effect. A phase III clinical trial in China with 110 metastatic melanoma patients (54 AM patients) showed that, compared with the dacarbazine group, the Endostar plus dacarbazine group had significant improvements in median PFS (4.5 months vs 1.5 months) and median OS (12.0 months vs 8.0 months) [91]. A phase II clinical trial in China with 29 patients (8 AM cases) evaluated the efficacy and safety of apatinib combined with temozolomide in advanced melanoma patients whose immunotherapy failed. The subgroup analysis showed a median PFS of 4.0 months and a median OS of 10.1 months in AM patients [92•]. Phase I trial of this regimen combined with anti-PD-1 treatment for AM is in progress (NCT04397770).

#### Targeted combination therapy

The efficacy of the combination of BRAF and MEK inhibitors in advanced AM patients has been described above. Notably, for melanomas with NRAS mutations, inhibition of MEK alone is not sufficient to completely inhibit the activation of downstream signaling mediated by NRAS through CDK4 [93]. Similarly, in vitro studies confirmed that overexpression of cyclins D1 and CDK4 mediates resistance to BRAF inhibitors [94]. A previous study confirmed that CDK4/6 inhibitors can overcome acquired resistance to BRAF/MEK inhibitors [95]. These results provided a theoretical basis for BRAF/MEK inhibitors combined with CDK4/6 inhibitors to treat advanced melanoma. However, another report showed that palbociclib combined with BRAF and MEK inhibitors might not work after BRAF inhibitor resistance is acquired [96].

## Future directions

Over the past decade, with the introduction of immune checkpoint inhibitors and targeted drugs, the prognosis of advanced/metastatic melanoma patients has dramatically improved. For example, their 5-year overall survival rate substantially rose from less than 10% to up to 40–50% [97]. However, due to the unique genomic and tumor immune microenvironment characteristics of AM, the efficacy of ICIs and targeted drugs in advanced AM patients is not ideal, and the treatment is still facing difficulties. Hence, exploring new treatment strategies is urgent.

### Immunotherapy

Acral melanoma has a unique tumor microenvironment, including relatively low expression of PD-L1 [98–100], a decreased number of tumor-infiltrating lymphocytes (TILs) [101–104], and a high neutrophil-lymphocyte ratio (NLR) [105]. This can also lead to poor efficacy of current ICI treatments in advanced AM patients. How to enhance a patient’s anti-tumor immune response by targeting new immune checkpoints as well as combination therapy might be the key to future immunotherapies.

#### Novel immune checkpoints

Due to the lack of studies with sufficient samples, the expression levels of immune checkpoints in the AM immune microenvironment remain unclear. Only one small-sample study showed that immune cells related to AM expressed multiple immune checkpoints, including PD-1, LAG-3, CTLA-4, VISTA, TIGIT, TIM-3, and ADORA2. Compared to CM, the expression of VISTA and ADORA2 was increased in AM, and the expression of TIGIT was similar. Although the expression of TIM-3 was significantly lower in AM than that in CM, it was still expressed in 29.2% of myeloid cells. For AM, VISTA, ADORA2, TIGIT, and TIM-3 might become novel immune checkpoints with research value in the future [106••].

Adenosine A2A receptor (ADORA2) can inhibit the accumulation of CD8^+^ T and NK cells in the tumor microenvironment [107–109], suggesting that ADORA2 might become a new immune checkpoint for AM treatment. Multiple antagonists of ADORA2 have been developed, with early indications that these compounds can effectively work combined with anti-PD-1 with responses in patients who derived no benefit from prior anti-PD-1/PD-L1 therapy [110].

VISTA (V-domain immunoglobin suppressor of T cell activation) is a type I transmembrane protein that has a high degree of structural homology with PD-L1 and is highly expressed in multiple immune cells [111, 112]. Preclinical studies have shown that inhibiting VISTA can inhibit the proliferation of CD4^+^ and CD8^+^ T cells [112]. The enhanced expression of VISTA in melanoma cells can also lead to increased PD-L1 expression in tumor-associated macrophages, increased Treg infiltration, and reduced MHC expression on dendritic cells [113]. Additionally, the expression of VISTA is related to acquired resistance to anti-PD-1 treatment [114, 115]. The results above indicated that the combination therapy of anti-VISTA and anti-PD-1 might be a potential and valuable treatment. For example, CA-170, a small-molecule dual antagonist of VISTA/PD-L1, is currently in phase I clinical trial (NCT02812875).

The T cell immunoreceptor with immunoglobulin and ITIM domain (TIGIT) is an inhibitory immune checkpoint upregulated in tumor antigen-specific CD8^+^ T cells and TILs of melanoma patients and is co-expressed with PD-1. TIGIT binds to two ligands, CD155 and CD112, that are expressed by melanoma cells and antigen-presenting cells to downregulate T and NK cell functions [116, 117]. Dual PD-1/TIGIT blockade also enhances the proliferation and function of tumor antigen-specific CD8^+^ T cells and TILs isolated from melanoma patients compared to single PD-1 blockade [118]. There is also evidence that increased expression of CD155 is associated with resistance to anti-PD-1 therapy [119]. Therefore, combining PD-1 and TIGIT inhibitors might be a potential strategy to treat AM patients. Currently, two related phase I/II studies are in progress (NCT04305041, NCT04305054).

The abnormal expression of T cell immunoglobulin and mucin domain 3 (TIM-3) in tumor tissues is often closely related to T cell depletion [120–124]. Previous studies have shown that, compared with T cells only expressing PD-1, T cells expressing both TIM-3 and PD-1 are more inhibited [125, 126]. Additionally, the upregulation of TIM-3 expression is related to the acquired resistance to anti-PD-1/PD-L1 treatment [127]. These results provided a theoretical basis for TIM-3 inhibitors combined with anti-PD-1/PD-L1 drugs in the treatment of advanced tumors. There are currently two ongoing clinical trials exploring the role of combined blockade of TIM-3 and PD-1 in advanced melanoma (NCT04139902, NCT04370704). However, multiple ligands of TIM-3 result in a single TIM-3 inhibitor that might not completely block the effects of TIM-3 [128]. Moreover, trametinib, a MEK inhibitor, can promote the expression of TIM-3, resulting in decreased CD8 + T cells, while anti-TIM-3 monoclonal antibody enhances anti-tumor immune function by stimulating CD8^+^ T cells, and reverses the depletion of T and NK cells in the trametinib-induced immune microenvironment. This suggested that trametinib combined with anti-TIM-3 drugs might also be a potential choice for melanoma treatments [129].

Additionally, the potential value of various new inhibitory immune checkpoints (BTLA [130–132], B7 family [133–137], IDO-1 [138, 139], LAG-3 [140, 141]) and costimulatory molecules (GITR [142–145], ICOS [146–148], OX40 [149–151], 4-1BB [152–154], CD40 [155, 156], CD27 [157–159]) has been confirmed for melanoma treatments. The efficacy and safety of some inhibitory checkpoints and costimulatory molecules in melanoma treatments, alone or combined with ICIs, have been preliminarily elucidated in early clinical trials [160–173] and others are in progress (NCT04773951, NCT04137900, NCT02554812). Currently, no large-sample study comparing the expression levels of these immune checkpoints in AM and CM tissues is available. Hence, the role of these immune checkpoints in AM treatments remains to be verified by further clinical trials.

#### Adoptive cell therapy

Adoptive cell therapy consists of the isolation of TILs from the excised tumor, expansion of these cells by interleukin-2 (IL-2) treatment, and reinfusion into lympho-depleted patients with IL-2 treatment. At present, although adoptive cell therapy (ACT) has achieved certain efficacy in advanced CM patients, there is a lack of clinical trials and retrospective analysis for AM patients to confirm its applicability in the treatment of advanced AM. Only a very small sample of clinical trials in Japan showed that this therapy might have a certain effect on advanced AM after treatment with ICIs [174].

#### Chimeric antigen receptor-engineered T cell therapy

The chimeric antigen receptor (CAR) structure consists of a single-chain variable fragment derived from a monoclonal antibody targeting a cancer-specific antigen, intracellular segment, signaling domain derived from TCR, and one or more co-stimulatory sequences (CD28, OX40, or 4-1BB) [175]. CAR can bind antigens to cancer cells and activate T cells [176]. CAR T-cell therapy had success in treating patients with hematologic diseases. However, little is known about its efficacy in AM patients.

GD2 gangliosides are sialic acid-containing glycosphingolipids that are overexpressed on several solid tumors, including melanoma [177]. A preclinical study with lesion samples from 288 melanoma patients evaluated the ability of anti-GD2/4-1BB CAR T cells to kill ganglioside GD2^+^ melanoma cells. Among the 288 samples, 49.3% (142/288) demonstrated positive staining for ganglioside GD2. Its expression was relatively more frequent in acral (50.0%) and mucosal (56.3%) melanomas than that in CSD (14.3%) and non-CSD (33.3%) melanomas. The median OS of patients exhibiting ganglioside GD2 expression was significantly shorter than those without ganglioside GD2 expression (31 months vs 47.1 months) [152]. This study provided a theoretical basis for the treatment of advanced AM with anti-GD2/4-1BB CAR T cells.

#### Vaccines

Vaccines for melanoma treatment might be used as antigen whole tumor cells, RNA or DNA, single or multiple peptides, or APCs displaying the target antigen [178]. At present, clinical trials of a large number of vaccines combined with ICIs in the treatment of advanced CM are in progress.

#### Tebentafusp

Tebentafusp is a bispecific protein consisting of a high-affinity T-cell receptor fused to an anti-CD3 effector that can redirect T cells to target glycoprotein 100-positive cells [179]. A phase III clinical trial showed that treatment with tebentafusp resulted in longer overall survival than therapy with single-agent pembrolizumab, ipilimumab, or dacarbazine among previously untreated patients with metastatic uveal melanoma [180]. Glycoprotein 100 (Gp-100) is a transmembrane glycoprotein, highly expressed in normal melanocytes and melanoma cells. HMB-45, which recognizes Glycoprotein 100 (Gp-100), has been repeatedly proven to be a sensitive and relatively specific marker of melanomas. Eighty percent of AMs stained with HMB-45 in a previous study [181]. Furthermore, tebentafusp potently activated antitumor immune responses in patients with metastatic melanoma [182]. These suggested that tebentafusp might be a potential choice for advanced AM treatment.

### Targeted therapy

#### Antibody-drug conjugates

Antibody-drug conjugates (ADCs) combine a monoclonal antibody (mAb) with a cytotoxic agent, allowing its specific delivery to targeted tumor cells overexpressing cognate tumor-associated antigens (TAAs) [183]. Previous studies have confirmed the antitumor activity and safety of ADCs such as glembatumumab vedotin and DEDN6526A in patients with advanced non-AM [184–187]. In addition, many clinical trials of ADC in the treatment of advanced melanoma are underway [188]. We look forward to the efficacy of ADCs in patients with advanced AM.

#### Emerging gene mutations

Besides low tumor mutational burden (TMB) [40] and low gene expression associated with antigen presentation and T cell inflammation [189, 190], AM has other unique genomic characteristics. Compared to CM, the frequency of BRAF (18% vs 46%) and RAS (21% vs 31%) mutations is lower in AM, but the frequency of NF1 mutation is higher (23% vs 10%), and the proportion of TWT is also higher (38% vs. 11%). Mutations in c-kit, NOTCH2, TYRP1, and PTEN, as well as oncogenic amplification of genes including TERT, CDK4, MDM2, CCND1, PAK1, and GAB2, are more common in AM than those in CM [191, 192••]. Novel targeted drugs targeting common mutations/amplification of AM might be potential targeted therapies for AM in the future.

Previous studies have shown that TERT aberrations are observed in 41% of AM patients, and in vitro TERT inhibition has cytotoxic effects on AM cells [193]. Another Chinese study showed that the incidence of TERT copy number gain in AM (61.5%) was higher than that in other melanoma subtypes [194], suggesting that TERT inhibition might be a potential therapeutic strategy for AM.

A study showed that 47.5% of all specimens presented an increase in the CCND1 copy number. This increase was associated with the Breslow thickness in AM [195]. This suggested that CCND1 might also be a potential target for AM treatment. NUAK2 participates in the regulation of proliferation and migration of melanoma cells by regulating the cell cycle [196]. A study showed that NUAK2 is negatively correlated with the OS and PFS of AM patients, and this effect is higher than that of CM [44], suggesting that NUAK2 has potential value for AM treatment. Previous studies have also reported that CDK4/6 inhibition can induce and maintain the T cell inflammatory microenvironment [197], and enhance the efficacy of PD-L1 checkpoint blockade [198, 199]. Additionally, genetic abnormalities in the CDK4 pathway are associated with innate resistance to anti-PD-1 therapy [200•]. These results provided a theoretical basis for CDK4/6 inhibitors combined with anti-PD-(L)1 antibody to treat advanced AM.

Cancer cells are often defective in DNA damage response and repair (DDR) and highly depend on other DNA repair pathways to avoid lethal DNA damage. Alterations in DDR genes have been shown to promote the expression of PD-L1, elevate the count of TILs, increase the TMB, and enhance immunogenicity via an increased neoantigen load, which are also potential determinants of the response to ICI treatments [201, 202]. Ataxia-telangiectasia and Rad3-related protein kinase (ATR) are essential components of DDR [203]. Recent clinical trials have confirmed that ceralasertib, an oral ATR inhibitor, combined with durvalumab/paclitaxel is effective in advanced melanoma (including AM) [204•, 205]. Moreover, PARP inhibitors have been proved to have therapeutic potential for tumors with DDR defects (including ATM and ATR mutations) [206, 207], and might also be used to treat advanced AM in the future. MDM2 can inhibit DRR by inhibiting p53 function [208]. The amplification of MDM2 is related to the excessive progression of tumors in metastatic AM patients after treatment with ICIs, suggesting that it might also become a potential target for advanced AM treatment in the future [209].

Furthermore, EP300 encodes the histone acetyltransferase paralogue p300 that manipulates different cellular processes (e.g., proliferation, apoptosis, and DNA repair) and promotes tumor growth through its downstream oncogene target MITF [210]. Recently, a study in China showed that, compared with other subtypes, EP300 copies in limb melanoma increased more frequently, and 30% (70/233) of AM lesion samples carried the copy number gains of the EP300-MITF axis. Additionally, AM with copy number gains of the EP300 pathway tended to be more aggressive [211]. Therefore, the EP300-MITF pathway might become a potential clinical target. Another study showed that the p300 inhibitor C646 can overcome the resistance of melanoma cells to BRAF inhibitors in vitro and in vivo, which also provides a theoretical basis for targeted combination therapy [212]. Additionally, HDAC inhibitors have been previously shown to inhibit the expression of MITF [213], and combined with nivolumab in the treatment of advanced melanoma it achieved good ORR [214], and related clinical trials are under recruitment (NCT04674683).

A preclinical study in China showed that the higher infiltration of cancer-associated fibroblasts was related to innate resistance of AM to PD-1 inhibitors, and the FAK inhibitor defactinib enhanced the efficacy of anti-PD-1 antibodies. This study provided a basis for the combination of FAK inhibitors and PD-1 inhibitors in the treatment of advanced AM [215].

The feasibility of using PI3K/Akt/mTOR inhibitors [216], ERK inhibitors [217], or WNT inhibitors [218] to treat AM is currently being investigated.

## Conclusions

At present, AM lacks effective intervention measures. The unique genomic and tumor immune microenvironment characteristics of AM are responsible for its poor response to current immunotherapy and other systemic treatments, and combined treatment is more likely to provide long-term clinical benefits. The clinical efficacy and safety of multiple combined therapies are currently being extensively studied. With an in-depth understanding of the genomic and tumor immune microenvironment characteristics of AM, it is possible to develop effective therapeutic drugs or methods and improve the survival of patients.

## Data Availability

Not applicable.
